# Cartilage Trauma Induces Necroptotic Chondrocyte Death and Expulsion of Cellular Contents

**DOI:** 10.3390/ijms21124204

**Published:** 2020-06-12

**Authors:** Josef Stolberg-Stolberg, Meike Sambale, Uwe Hansen, Alexandra Schäfer, Michael Raschke, Jessica Bertrand, Thomas Pap, Joanna Sherwood

**Affiliations:** 1 Institute for Musculoskeletal Medicine, University Hospital Muenster, Albert-Schweitzer-Campus 1, Building D 3, 48149 Muenster, Germany; Meike.Sambale@ukmuenster.de (M.S.); Uwe.Hansen@ukmuenster.de (U.H.); Alexandra.Schaefer@ukmuenster.de (A.S.); thomas.pap@ukmuenster.de (T.P.); sherwood@uni-muenster.de (J.S.); 2 Department of Trauma-, Hand- and Reconstructive Surgery, University Hospital Muenster, Albert-Schweitzer-Campus 1, Building W1, 48149 Muenster, Germany; michael.raschke@ukmuenster.de; 3 Department of Orthopaedic Surgery, Otto-von-Guericke University, Leipziger Straße 44, 39120 Magdeburg, Germany; jessica.bertrand@med.ovgu.de

**Keywords:** necroptosis, post-traumatic arthritis, cell death, inflammation, articular cartilage

## Abstract

Necroptotic cell death is characterized by an activation of RIPK3 and MLKL that leads to plasma membrane permeabilization and the release of immunostimulatory cellular contents. High levels of chondrocyte death occur following intra-articular trauma, which frequently leads to post-traumatic osteoarthritis development. The aim of this study is to assess necroptosis levels in cartilage post-trauma and to examine whether chondrocyte necroptotic mechanisms may be investigated and modified in vitro. Fractured human and murine cartilage, analysed immunohistochemically for necroptosis marker expression, demonstrated significantly higher levels of RIPK3 and phospho-MLKL than uninjured controls. Primary murine chondrocytes stimulated in vitro with the TNFα and AKT-inhibitor alongside the pan-caspase inhibitor Z-VAD-fmk exhibited a significant loss of metabolic activity and viability, accompanied by an increase in MLKL phosphorylation, which was rescued by further treatment of chondrocytes with necrostatin-1. Transmission electron microscopy demonstrated morphological features of necroptosis in chondrocytes following TNFα and Z-VAD-fmk treatment. Release of dsDNA from necroptotic chondrocytes was found to be significantly increased compared to controls. This study demonstrates that cartilage trauma leads to a high prevalence of necroptotic chondrocyte death, which can be induced and inhibited in vitro, indicating that both necroptosis and its consequential release of immunostimulatory cellular contents are potential therapeutic targets in post-traumatic arthritis treatment.

## 1. Introduction

Post-traumatic arthritis (PTA) can develop following a variety of joint injuries but most predictably after intra-articular fracture. The current treatment standard includes open reduction and internal fixation to restore joint congruity. However, approximately 12% of 21 million U.S. Americans suffering from osteoarthritis (OA) have an aetiology of previous joint trauma [1]. While recent research identified an early inflammatory phase including chondrocyte death, release of cytokines and damage-associated molecular pattern molecules (DAMPs), so far there are no approved therapies to prevent the progression of traumatic joint injury into the chronic disease phase characterized by pain, cartilage degeneration, and joint dysfunction [2,3].

Chondrocyte death occurs after cartilage trauma, which has been shown in various ex vivo models and in human osteochondral fragments collected from joint trauma patients [4,5]. Resulting hypocellularity has been suggested to predispose patients to matrix degeneration and eventual development of OA [6,7]. However, the amount of apoptotic chondrocytes, empty lacunae, and apoptotic bodies found in OA cartilage varies greatly between studies, from 0.1–88%, and thus the role of apoptosis remains controversial [8,9,10]. With respect to PTA, the amount of cell death depends on the loading magnitude [11,12,13]. Immediate cell death is generally termed necrosis, occurring along fracture edges and after high-energy impact trauma, while low-energy loaded areas seem to be affected primarily by apoptosis [14,15]. As compelling concepts explaining the inflammatory aspect of immunologically silent apoptosis after joint trauma are missing, other forms of cell death need to be investigated [9].

Necroptosis, also termed ‘programmed necrosis’, is mediated by the necrosome consisting of receptor interacting serine/threonine protein kinase 1 and 3 (RIPK1 and RIPK3) and the pseudokinase mixed-lineage domain-like protein (MLKL) [16,17]. Upon activation, phosphorylated MLKL is localized to the plasma membrane, ultimately resulting in cell rupture [18,19]. Within the field of cartilage biology, Roach et al. defined the term chondroptosis as apoptosis in a non-classical manner characterized by patchy chromatin, increased rough endoplasmatic reticulum (rER), Golgi apparatus, vacuoles, and final expulsion of cell remnants into the lacunae [20]. Similar chondrocyte morphology was described in diseases such as alkaptonuria and chondrocalcinosis, but without elaboration of the molecular pathways involved [21,22]. Caspase-independent necroptotic cell death was first analysed in chondrocytes with the pseudoachondroplasia-inked mutation of the cartilage oligomeric matrix protein gene, and so far only leptin has been identified as a factor able to protect chondrocytes from TNFα-induced necroptosis [23,24]. In vivo inhibition of both necroptosis and apoptosis has been shown to attenuate mechanical force-mediated cartilage thinning [25,26,27]. This study presents further evidence demonstrating necroptotic activity in articular cartilage from human PTA patients.

Necroptosis is considered to be highly immunogenically active and is often mediated by the release of DAMPs including nucleic acids [28,29]. Matching pattern-recognition receptors (PRRs) such as Toll-like receptor 3 (TLR3) have been found to mediate inflammatory changes, e.g., through Il-33 and MMP-3 [30,31]. This is supported by recent findings stating that the catabolic activity of live chondrocytes actively contributes to arthritic changes, rather than only the reduced cartilage matrix turnover caused by death of chondrocytes [32,33]. Thus, re-defining the role of chondrocyte death in the inflammatory phase following joint trauma and in subsequent PTA development requires the study of immunogenically active forms of chondrocyte death.

The objectives of this study are to assess the presence of necroptotic chondrocytes after intra-articular fracture, analyse whether chondrocyte necroptosis may be induced in vitro, and to investigate whether DAMP release following necroptosis may act as a trigger for further inflammation within the joint.

## 2. Results

Necroptosis can be detected in human samples collected after intra-articular fracture, whilst cartilage injury induces chondrocyte necroptosis in a murine ex vivo trauma model.

**Figure 1:**
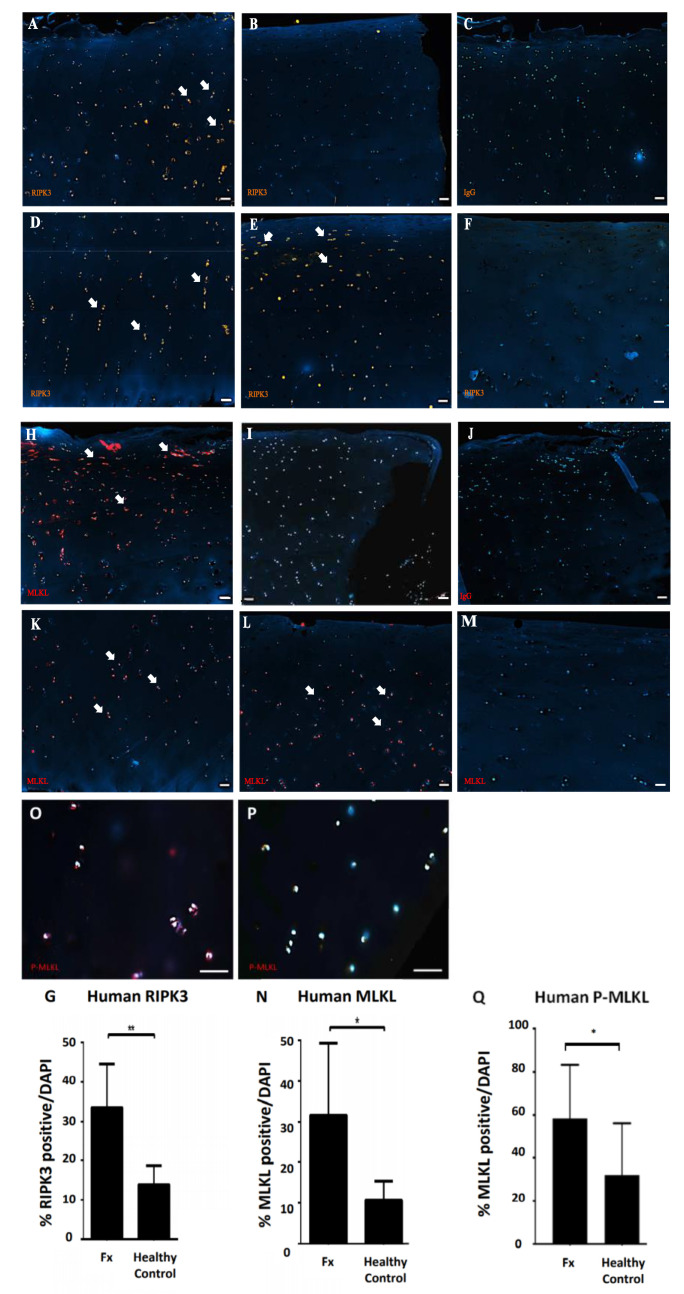
Immunofluorescence staining of (**A**–**F**) receptor interacting serine/threonine protein kinase-3 (RIPK3) and (**H**–**M**) pseudokinase mixed-lineage domain-like protein (MLKL) positive chondrocytes in human intra-articular fracture cartilage shows (**A**,**H**) positive chondrocytes mostly clustered in areas not immediately adjacent to the fracture edge (arrows) and (**B**,**I**) rare positive chondrocytes immediately adjacent to the fracture edge. (**C**,**J**) No signal is observed in respective IgG controls. (**D**,**K**) Few chondrocytes show RIPK3 or MLKL expression in the deep zone (arrows), while (**E**,**L**) both RIPK3 and MLKL positively stained chondrocytes are found mainly within the superficial and middle zones (arrows) (Scale bar 200 μm). (**F**,**M**) Healthy control cartilage shows no pattern of positively stained cells (Scale bar 100 μm). (**G**,**N**) Quantification of RIPK3- and MLKL-positive chondrocytes in fractured (*n* = 7) compared to healthy (*n* = 6) human samples (** *p* < 0.01). Immunofluorescence staining of phospho-MLKL-positive chondrocytes in intra-articular fractured (**O**) and uninjured human samples (**P**) (Scale bar 50 μm) demonstrates significantly higher levels of MLKL phosphorylation in chondrocytes in fractured cartilage (**Q**) (* *p* < 0.05).

The necroptosis markers RIPK3 and MLKL were identified by immunostaining within human intra-articular fracture cartilage analysed 6.7 (±4.5) days following initial injury (Table 1). Both RIPK3- and MLKL-positive chondrocytes were found to be predominantly located within chondrocyte clusters in sites away from the fracture edge (Figure 1A,H). Chondrocytes directly at the fracture edge were rarely found to be positive for either RIPK3 or MLKL (Figure 1B,I), while no positive cells were found in IgG controls (Figure 1C,J).

RIPK3- and MLKL-positive cells were rarely found within the cartilage deep zone or near the calcification tidemark (Figure 1D,K), but instead were typically limited to the upper cartilage layer (Figure 1E,L). Healthy control cartilage rarely contained RIPK3- (Figure 1F) and MLKL- (Figure 1M) positive cells with no recurring patterns observed. In total, human fractured samples contained an average of 32.34% (±10.6%) RIPK3-positive and 31.67% (±16.34%) MLKL-positive chondrocytes compared to control samples that had an average of 13.94% (±4.37%) RIPK3-positive (Figure 1G; *p* < 0.01) and 10.72% (±4.28%) MLKL-positive chondrocytes (Figure 1N; *p* < 0.05).

To directly assess the activity of necroptotic MLKL signalling, phosphorylated-MLKL-positive chondrocytes were detected in fractured (Figure 1O) and healthy (Figure 1P) human cartilage samples. Quantification showed 58.5% (±23.3%) p-MLKL-positive chondrocytes in fracture samples whereas non-OA control samples had an average of 32.2% (±23.1%) positive p-MLKL cells (Figure 1Q; *p* < 0.05) demonstrating an increased activity of the RIPK/MLKL necroptotic signalling pathway within cartilage samples from intra-articular fracture patients compared to healthy controls.

**Figure 2:**
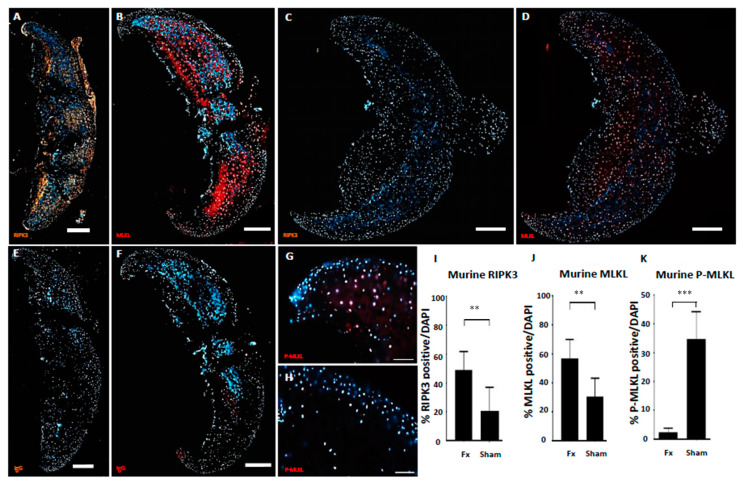
Fractured hip cartilage of skeletally mature mice shows more RIPK3- (**A**) and MLKL- (**B**) positive chondrocytes compared to uninjured (**C**,**D**) and IgG controls (**E**,**F**) (Scale bar 200 μm). p-MLKL staining on fractured (**G**) and uninjured (**H**) cartilage (Scale bar 50 μm). Quantification of RIPK3-positive (**I**), MLKL-positive (**J**) and p-MLKL-positive chondrocytes normalised for DAPI-positive cells, (**K**) demonstrating significantly more necroptotic chondrocytes within the injured samples as compared to uninjured controls (** *p* < 0.01, *** *p* < 0.001, *n* = 7).

In order to create an intra-articular fracture scenario ex vivo, murine hip caps obtained from 6-week-old mice were fractured using a pistil and left in culture for 24 h before analysis by immunostaining for necroptotic marker expression. RIPK3 and MLKL positively stained chondrocytes were predominantly identified in close proximity to the site of fracture, whilst cells at the immediate edge of the fracture site and in relatively unaffected areas were rarely positive for necroptotic markers (Figure 2A,B). Murine cartilage samples that underwent ex vivo fracture showed 48.93% (±12.9%) RIPK3-positive (Figure 2I) and 56.8% (±13.2%) MLKL-positive chondrocytes as opposed to 20.76% (±16.23%) and 30.73% (±12.55%), respectively, in unchallenged controls (Figure 2J; *p* < 0.01).

Activation activity of MLKL was again measured by immunostaining for phosphorylated MLKL. p-MLKL-positive chondrocytes were observed at high frequency in fractured hipcaps (Figure 2G), while cellular staining in sham controls was found to be low (Figure 2H). Quantification of staining showed 24.7% (±8.9%) p-MLKL-positive chondrocytes in fractured hipcaps compared to 2.5% (±1.3%) in uninjured contralateral controls (Figure 2K; *p* < 0.001), demonstrating that the RIPK3/MLKL necroptotic pathway can be induced by direct trauma and measured during ex vivo fracture of murine hip cartilage.

### 2.1. Necroptotic Cell Death Can Be Induced In Vitro and Inhibited by Necrostatin-1

**Figure 3:**
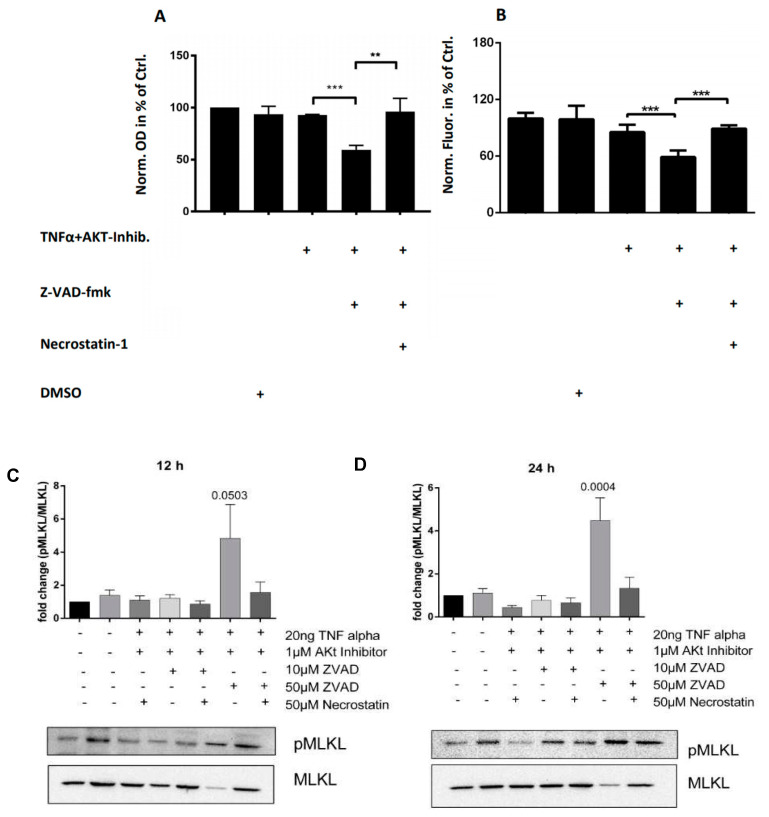
(**A**) 3-(4,5-dimethylthiazol-2-yl)-2,5-diphenyltetrazolium bromide (MTT) assay reductase activity (OD) demonstrates a significant decrease in cell metabolic activity in TNFα + AKT-inhibitor + Z-VAD-fmk-treated chondrocytes compared to controls, which can be partly inhibited by RIPK1-inhibitor necrostatin-1. (**B**) Cell TiterGlo Luminescence assay measurement of ATP concentration (fluorescence) demonstrates a significant decrease in chondrocyte viability in TNFα + AKT-inhibitor + Z-VAD-fmk-treated chondrocytes compared to controls, which can be partly inhibited by RIPK1-inhibitor necrostatin-1. Quantified Western blot analysis of phospho-MLKL levels demonstrates increased MLKL activity following both 12 h (**C**) and 24 h (**D**) stimulation with TNFα + AKT-inhibitor + Z-VAD-fmk that could be reversed using necrostation-1. All data presented are representative of at least three independent biological samples analysed. (** *p* < 0.01, *** *p* < 0.001)

In order to establish an in vitro system for investigating necroptosis in chondrocytes, murine primary chondrocytes were stimulated with TNFα alongside an AKT-inhibitor. When the pan-caspase inhibitor Z-VAD-fmk was additionally added to block the apoptotic pathway, a significant decrease in metabolic activity was observed by 3-(4,5-dimethylthiazol-2-yl)-2,5-diphenyltetrazolium bromide (MTT) assay from 92.596% (±0.39%) in TNFα + AKT-inhibitor-treated chondrocytes to 59.49% (±3.37) in TNFα + AKT-inhibitor + Z-VAD-fmk-treated chondrocytes (Figure 3A, *p* < 0.001)). When the RIPK1 inhibitor necrostatin-1 was added to TNFα + AKT-inhibitor + Z-VAD-fmk-treated chondrocytes, cell metabolic levels increased to the level of TNFα + AKT-inhibitor-treated chondrocytes, indicating that necrostatin-1 may be used to block the necroptosis driven loss of metabolic activity in chondrocytes (increased to 96.09% (±10.46%) of the control (*p* < 0.01)).

To confirm a loss of viability in chondrocytes undergoing necroptosis induction in vitro, chondrocytes treated with either the TNFα + AKT-inhibitor or TNFα + AKT-inhibitor + Z-VAD-fmk were subjected to the CellTiter Glo^®^ assay. Caspase inhibition using Z-VAD-fmk in TNFα + AKT-inhibitor-treated chondrocytes decreased luminescence from 85.42% (±7.14%) to 58.98% (±6.53%) of the control (Figure 3B, *p* < 0.001). The addition of the RIPK1 inhibitor necrostatin-1 increased viability to 89.23% (±3.28) of controls (*p* < 0.001), demonstrating a rescue of viability through additional inhibition of the RIPK pathway.

To examine whether markers of necroptosis detected within both human and murine cartilage fracture samples can also be observed within our in vitro induction system, MLKL phosphorylation in treated chondrocytes was measured by Western blot; 12 h following stimulation with TNFα + AKT-inhibitor + Z-VAD-fmk, p-MLKL levels were found to increase in comparison to both controls and to necrostatin-1-treated chondrocytes (Figure 3C; *p* = 0.0503). After 24 h stimulation, MLKL phosphorylation was significantly increased in TNFα + AKT-inhibitor + Z-VAD-fmk-treated chondrocytes, which was again inhibited by necrostatin-1 (Figure 3D; *p* < 0.05). No significant differences were found when chondrocytes were stimulated with TNFa + AKT-inhibitor with or without necrostatin-1 (*p* > 0.05). These data show that the loss of metabolic activity and viability observed in TNFα + AKT-inhibitor + Z-VAD-fmk-treated chondrocytes is accompanied by a significant increase in MLKL phosphorylation that can be decreased via RIPK1 inhibition, indicative of a specific induction of necroptosis in chondrocytes in our experimental set up.

### 2.2. Necroptotic Chondrocytes Contain Exocytotic Vesicles and Release Nucleic Acids Which Are Likely to Act as Ligands for Pattern Recognition Receptors

**Figure 4:**
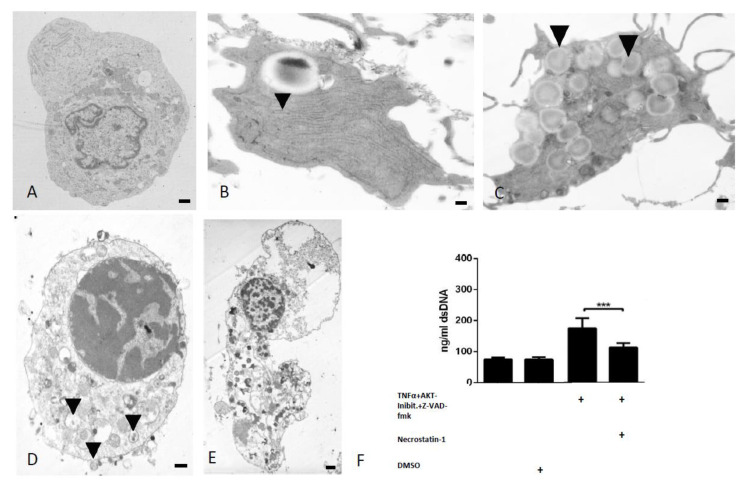
(**A**) TEM images show no pathologic cellular morphology in control samples. Morphological characteristics of necroptotic cell death including increased rER (**B**, indicated by arrows), autophagic vacuoles (**C**, indicated by arrows), exocytotic vesicles (**D**, indicated by arrows), and final cell disintegration (**E**) (Scale bar 400 nm). (**F**) A significant increase in dsDNA release by chondrocytes stimulated with Z-VAD-fmk in addition to the TNFα + AKT-inhibitor is observed compared to controls, which can be reduced following treatment with necrostain-1 (*** *p* < 0.001, *n* = 7).

While no distinct features were observed by transmission electron microscopy within our untreated controls (Figure 4A), TNFα + AKT-inhibitor + Z-VAD-fmk-stimulated ‘necroptotic’ chondrocytes exhibited many morphological hallmarks associated with necroptosis. Features frequently found include increased rough endoplasmic reticula and Golgi apparatus (Figure 4B), large autophagic vacuoles (Figure 4C), extrusions of cellular material into extracellular space (Figure 4D), and final disintegration with the release of cell remnants (Figure 4E) [20].

In order to assess whether in vitro induced necroptosis leads to the release of dsDNA, which may act as a DAMP further exacerbating cartilage catabolism, the concentration of dsDNA released into the supernatant of chondrocytes following necroptosis induction was quantified. TNFα + AKT-inhibitor-stimulated chondrocytes released an average concentration of 100.4 ng/mL (±6.18 ng/mL) dsDNA, showing no significant difference compared to vehicle-treated controls (Figure 4F; *p* > 0.05). However, inhibiting apoptosis by adding Z-VAD-fmk led to a significant increase in dsDNA release to 175.19 ng/mL (±30.26 ng/mL) (*p* < 0.001). When necroptosis was additionally inhibited using necrostatin-1, dsDNA release was again decreased to 113.14 ng/mL (±13.18 ng/mL), suggesting that the specific activation of necroptosis in chondrocytes leads to the release of dsDNA (Figure 4F; *p* < 0.001).

## 3. Discussion

The aims of this study were to address the presence of necroptotic chondrocytes after intra-articular fracture, to examine whether chondrocyte necroptosis may be induced in vitro and to investigate whether cellular components are released during necroptosis, which may act as a possible trigger for inflammation within the joint.

The role of chondrocyte death in PTA and OA is a matter of continuous debate [9,15]. Initially, research focused on the study of apoptosis in cartilage; however, results of TUNEL staining-based studies reported vast differences in the relative numbers of apoptotic cells found in samples [8,34]. Indeed, the very rare reported presence of true apoptotic bodies within an avascular tissue such as articular cartilage puts an additional question mark behind the concept of necrosis and apoptosis as the only modes of cell death in cartilage [8]. The facts that the TUNEL assay is not able to distinguish between necroptosis and apoptosis and that necroptosis results in a final plasma membrane rupture suggests that some of in the past TUNEL-positive described chondrocytes may rather have been necroptotic cells [16,35]. Later, the concepts of dark chondrocytes and chondroptosis were introduced by Roach et al. as a purely morphological analysis [20]. Mechanistically, it is known only that these cells are TUNEL positive, with some authors suggesting caspase involvement [9,36,37]. Meanwhile, recent research has aimed to characterize necroptotic cells in terms of their morphological description with features such as swelling of organelles, generation of extracellular vesicles, and plasma membrane permeabilization and collapse frequently described [35,38,39]. Furthermore, chondrocyte necroptosis has been shown in human primary OA cartilage samples and is associated with PGE2 and NO release [40]. However, without specific molecular markers for chondroptosis, it remains difficult to accurately assess the role of “non-classical apoptosis” in cartilage biology, and evidence for the presence on PTA is lacking in the literature.

While necroptosis-inducing mechanisms including via the TNF receptor 1 (TNFR1) and Toll-like receptor-3 and -4 pathways have been identified, the specific role of necroptosis in health and disease remains the subject of current research [16,17]. In chondrocytes, TNFR1 stimulation is not cytotoxic but pro-inflammatory [41]. While the literature describes additional TNFR1 Complex I destabilization by inhibitor of apoptosis (IAP), transforming growth factor beta-activated kinase 2 (TAK1) or NFκB essential modulator (NEMO) inhibition as essential for necrosome formation and subsequent necroptosis, authors frequently use the protein synthesis inhibitor cylcoheximide [40]. However, the mechanisms behind TNFα + cycloheximide-induced necroptosis remain unclear and could not be reproduced in this study [16,25]. Instead, we inhibited AKT which is described in detail to promote cell survival via caspase-9, BAD, and FoxO transcription factor down-regulation [42,43]. Furthermore, our data show the specific inhibition of necroptosis following treatment with the RIPK1 inhibitor necrostatin-1. Although recent literature also describes RIPK1-independent necroptosis, the combination of assessing RIPK3, MLKL, and p-MLKL expression and responsiveness to necrostatin-1 treatment is viewed as the gold standard for detecting necroptosis and is regarded as superior in accuracy compared to early studies addressing the absence of caspase-3 and presence of necrotic morphology, which cannot distinguish between different types of necrotic cell death [16,44].

The discussion on chondrocyte death as a cause or consequence of OA has recently been fueled by findings indicating that early cell death ameliorates disease progression [32,33]. Additionally, Heinemeier et al. used C-14 radiocarbon dating of healthy as well as OA cartilage and showed that virtually no collagen turnover occurs after skeletal maturity, suggesting that the role of chondrocytes in cartilage matrix maintenance is limited [45]. While these data indicate that abnormally activated cells contribute to inflammation and tissue damage in OA, chondrocytes might behave differently following a sudden event such as an intra-articular fracture. Our murine trauma samples show a significant number of necroptotic chondrocytes present 24 h after trauma. The advantage of our use of healthy murine cartilage was that a standardized facture of consistent force was used, avoiding variation caused by individual patient factors found when using human explants [40]. Although the human cartilage presented fewer positively stained chondrocytes, particularly at the fracture edges, which might be caused by the variability of trauma mechanism and time point of tissue fixation, a boost in inflammatory markers might be an explanation for early progression of clinical PTA [3,46]. Necroptosis-associated mediators include Il-33, Il-1α and DAMPs such as HMGB1 and dsDNA [28,29,47]. Focusing on free nucleic acids, we could show an increased concentration within the supernatant of our necroptotic cells. This released dsDNA has the ability to bind PRRs such as TLR3, perpetuating inflammation and further cell death [30,31]. However, a full analysis of the gene and protein expression profile of chondrocytes undergoing necroptosis would advance our understanding of both the mechanism of necroptosis and of the alterations to anabolic and catabolic processes during chondrocyte necroptosis that may also exacerbate cartilage destruction. A limitation of this study is the lack of analysis of apoptotic markers and quantification of alternative forms of cell death, including apoptosis. Zhang et al. induced mandibular PTA and found both necroptosis and apoptosis present in PTA [25]. Riegger et al. found cleaved caspase 8-positive cells predominantly in the superficial zone, while p-MLKL-positive cells were instead located in the deep zone of primary OA samples [40]. Thus, future clinical research should address necroptosis and apoptosis inhibition as well as the suppression of joint inflammation in intra-articular fracture patients in order to prevent development of PTA.

In conclusion, our data identify the presence of necroptotic chondrocytes in fractured human and murine cartilage. Furthermore, we are able to demonstrate necroptosis induction and its inhibition in vitro, alongside the increase of dsDNA release from chondrocytes undergoing necroptosis. Targeting post-traumatic chondrocyte necroptosis and subsequent DAMP release may be an important treatment strategy for the prevention of PTA development in joint trauma patients.

## 4. Materials and Methods

### 4.1. Human Cartilage Fragments

Osteochondral fragments (*n* = 7) from patients undergoing open reduction and internal fixation of an intra-articular fracture were collected at the time of surgery (Table 1). All samples were not required for surgical reconstruction und would otherwise have been discarded (ethics committee of the Medical Association of Westfalen-Lippe, no: 2014-365-f-S, 28 July 2014). Full thickness control cartilage (*n* = 6) was obtained from the Centre of Pathology and Forensic Medicine in Magdeburg (Institutional Review Board of the Medical School IRB: 23/16, Otto-von-Guericke University, Magdeburg, Germany) from patients with no macroscopic arthritic changes and no visible joint trauma or onset of decay. All samples were fixed in 4% neutral buffered formalin, decalcified, dehydrated, and embedded in paraffin.

### 4.2. Murine Cartilage Explants

Six-week-old wild type mice were euthanized, and femoral heads were removed under aseptic conditions (State Agency for Nature, Environment and Consumer Protection North Rhine-Westphalia, Germany, Project Number 84-02.05.50.15.005). Cartilage derived from the left hip (*n* = 7) was fractured once using a pistil, while cartilage from the right hip (*n* = 7) was left untreated. Fractured (fx) and unfractured (sham) samples were incubated for 24 h in supplemented DMEM medium at 37 °C and 5% CO_2_. Samples were fixed in 4% Paraformaldehyde at 4 °C overnight, dehydrated, and embedded in paraffin.

### 4.3. Immunohistochemistry

Tissue sections (8 μm) were deparaffinised, rehydrated, and subjected to digestion with either 1 × trypsin/EDTA (Sigma-Aldrich, Taufkirchen, Germany) for 20 min (murine samples) or 1500 U/mL pepsin (Sigma-Aldrich, St. Louis, MA, USA) for 45 min (human samples) at 37 °C. All sections were incubated overnight with anti-MLKL (orb32399, Biorbyt, Cambridge, UK) or anti-RIPK3 antibody (ab56164, Abcam, Cambridge, MA, USA) or IgG (Cell Signaling, #3900s, Danvers, MA, USA) at a concentration of 1:200 at 4 °C. For phospho-MLKL (p-MLKL) staining, the sections were treated with citrate buffer, pH 6, and were incubated with p-MLKL (ab196436, Abcam, Cambridge, MA, USA) at a concentration of 1:150 at 4 °C. Subsequently, samples were labelled with DAPI and secondary antibody (Alexa Fluor 546 A10040, Thermo Fisher Scientific, Waltham, MA, USA) before imaging. MLKL- and RIPK3-positive chondrocytes were counted and expressed as a percentage of DAPI-stained nuclei.

### 4.4. Chondrocyte Isolation

Primary chondrocytes were isolated from costal cartilage of 8- to 12-week-old C56Bl/6J mice. Cartilage was rinsed twice in phosphate-buffered saline (PBS) and placed into digestion solution containing 2 mg/mL collagenase type 4 (Worthington Biochemical Corporation, Lakewood, NJ, USA) and 4% penicillin (10,000 U/mL)/streptomycin (10 mg/mL) for 4 h. After tissue agitation and detachment of remaining soft tissue, cartilage was incubated in digestion solution in a thermal incubator under 5% CO_2_ and 37 °C overnight. For all experiments, P0 cells were cultured in Dulbecco’s Modified Eagle’s Medium (DMEM) containing 10% fetal bovine serum, 1 mM sodium pyruvate, and 1% penicillin/streptomycin at a density of 3.2 × 10^4^ chondrocytes cm^-2^ for 24 h before treatment. Chondrocyte morphology, including rounded/polygonal shape and granular cytoplasm, was confirmed under light microscope.

### 4.5. Induction of Necroptosis in In Vitro Chondrocytes

P0 chondrocytes cultured for 24 h in 96-well plates were divided into five groups: Control, DMSO control, 20 ng/mL murine Tumor Necrosis Factor-α (R&D Systems) + 1 μM AKT-inhibitor (Merck Chemicals, Darmstadt, Germany) with or without 50 μM pan-caspase inhibitor Z-VAD-fmk (Selleckchem, Munich, Germany). Where indicated, 50 μM RIPK1-inhibitor Necrostatin-1 (Selleckchem, Munich, Germany) was added (*n* = 3). For CellTiter Glo^®^ assay (*n* = 7) and transmission electron microscopy, the addition of 10 μM Z-VAD-fmk was sufficient to induce significant changes. Chondrocytes were incubated for 2 h with the inhibitors only, followed by stimulation with the ligand together with the inhibitors. 

### 4.6. Viability Assessment

After 24 h of stimulation, the MTT (Sigma-Aldrich, St. Louis, MO, USA) and CellTiter Glo^®^ 2.0 Assay (Promega, Madison, WI, USA) were used according to the manufacturer’s instructions. Absorbance (570 nm) and luminescence were measured using a micro-plate reader (Tecan, Crailsheim, Germany). Cell supernatants were collected for further experiments.

### 4.7. SDS-PAGE and Western Blotting

Murine chondrocytes were stimulated as indicated above. After 12 or 24 h, the cells were washed once in ice-cold PBS. Total cell extracts were obtained by scraping the cells in extraction buffer (10 mM Hepes, 1.5 mM MgCl2, 10 mM KCl, 0.5 mM DTT, and 0.05% NP-40, pH 7.9) containing phosphatase and protease inhibitors (Roche) and leaving the lysates on ice for 30 min. The protein extracts were run on an SDS-PAGE and transferred to a nitrocellulose membrane (GE Healthcare), which was blocked in blocking buffer (5% BSA containing 1% Tween20). The following primary antibodies were used: p-MLKL (1:1000, ab196436, Abcam) or anti-MLKL (1:1000, orb32399, Biorbyt, Cambridge, UK) as a loading control. Goat-anti rabbit HRP (1:8000, cell signalling # 7074) was used as secondary antibody, and proteins were detected using ECL-solution (Abcam, ab133406).

### 4.8. Transmission Electron Microscopy

Primary murine chondrocytes were stimulated for 12 h und subsequently fixed in 100 mM cacodylate buffer (pH7.4) containing 2.5% (*v*/*v*) glutaraldehyde and 2% (*v*/*v*) formaldehyde at 4 °C overnight. Cells were washed with PBS and post-fixed in 0.5% (*v*/*v*) osmium tetroxide and 1%(*w*/*v*) potassium hexacyanoferrate (III) in 0.1 M cacodylate buffer for 2 h at 4 °C followed by washing with distilled water. After dehydration in an ascending ethanol series, cells were incubated with propylene oxide (2 × 15 min) and embedded in EPON using BEEM capsules. Ultrathin sections were collected on copper grids and were negatively stained with 2% uranyl acetate for 10 min. Electron micrographs were taken at 60 kV with a Phillips EM-410 electron microscope using imaging plates (Ditabis, Pforzheim, Germany).

### 4.9. PicoGreen Assay

A Quant-iT™ dsDNA broad-range kit (Quant-iT™ PicoGreen™ dsDNA Assay Kit, Molecular Probes^®^, Oregon, USA) was used according to manufacturer’s instructions. Briefly, 50 μL of supernatants collected from chondrocytes stimulated as described and standards (Lambda DNA standard) were added to a 96-well plate. Quant-iT™ reagent was diluted 1:200 in TE buffer (10 mM Tris-HCl, 1mM EDTA, pH 7.5), and 100 μL was added to each well. Fluorescence (excitation/emission 480/520 nm) was measured after 10 min incubation using a micro-plate reader.

### 4.10. Statistics

GraphPad Prism Software V.5.0.c (GraphPad Software Inc, San Diego, CA, USA) was used to perform Student’s *t*-tests for single comparisons of immunohistochemistry, a one-way ANOVA for western blot, and Tukey’s multiple comparisons test for all other analyses. Data are presented as the mean ± SD, with *p* < 0.05 determining the level of significant difference. * *p* < 0.05; ** *p* < 0.01; *** *p* < 0.001.

## Figures and Tables

**Table 1:** Patient characteristics of analysed human samples.

Age	Sex	Location	Classification	Days after Trauma	Years Follow-Up	Kellgren & Lawrence Grade
66	f	radial head fracture	Mason II	3	1	I
51	m	talus fracture	Marti/Weber I	12	3	II
53	m	tibia plateau fracture	Moore V	4	3	IV
61	m	talus fracture	Marti & Weber I	9	3	IV
95	f	olecranon fracture	Schatzker D	3	-	-
62	m	radial head fracture	Mason III	2	1	I
33	m	cuboid fracture	Chopart Fracture	14	2	II
